# ADA-YOLO: An Adaptive Dynamic Aggregation Network for Small Object Detection in UAV Imagery

**DOI:** 10.3390/s26123908

**Published:** 2026-06-19

**Authors:** Jiajun Chen, Shaochen Jiang, Yongming Li, Sulaiman Tuersunayi, Yong Liu

**Affiliations:** College of Computer Science and Technology, Xinjiang University, Urumqi 830046, China; 107552403772@stu.xju.edu.cn (J.C.); lym@xju.edu.cn (Y.L.); tursunay@stu.xju.edu.cn (S.T.); 107552403819@stu.xju.edu.cn (Y.L.)

**Keywords:** UAV small object detection, YOLO, P2 detection head, dynamic upsampling, adaptive feature fusion

## Abstract

Unmanned Aerial Vehicle (UAV) image object detection holds significant application value in the low-altitude economy, traffic monitoring, intelligent agriculture, and disaster rescue. However, due to the top-down perspective, UAV images typically suffer from challenges such as small target scales, dense object distribution, severe occlusions, and complex backgrounds. These issues often limit the recall and localization accuracy of general-purpose detectors when they are directly applied to UAV small-object detection scenarios. To address these aforementioned challenges, this paper proposes an Adaptive Dynamic Aggregation YOLO network, termed ADA-YOLO. The novelty of ADA-YOLO lies in its highly efficient combinatorial design specifically tailored for UAV small object detection, while retaining the efficient backbone of YOLOv8, we systematically reconstruct the neck and detection head to improve accuracy. Specifically, a high-resolution P2 detection branch is incorporated to construct a P2–P5 multi-scale prediction structure. Furthermore, the lightweight DySample dynamic upsampling module is adopted to replace traditional upsampling methods, and an Adaptive Spatial Feature Fusion (ASFF) mechanism is introduced to alleviate semantic conflicts and noise interference during multi-scale feature fusion. This synergistic combination explicitly addresses multi-scale representation challenges and enhances small-object detection performance in complex scenes. Comparative experiments with the baseline YOLOv8n on the VisDrone2019 dataset demonstrate that ADA-YOLO achieves an improvement of 11.3% in mAP@0.5 and 8.2% in mAP@0.5:0.95. The improved model achieves these performance gains with a modest parameter increase and acceptable computational complexity. Finally, ablation experiments further validate the effectiveness of each individual module and their synergistic gains.

## 1. Introduction

Over the past few years, unmanned aerial vehicles (UAVs) have experienced extensive deployment across diverse domains, including traffic surveillance, smart agriculture, and emergency rescue operations, primarily driven by their exceptional maneuverability and expansive observational range. Object detection, as a key task in UAV visual perception systems, directly influences the effectiveness of subsequent tracking, identification, and decision-making. However, compared with natural scene images, UAV aerial image object detection faces greater challenges [1,2,3]. First, the high-altitude top-down viewpoint causes a large number of targets to occupy very few pixels, making detailed information prone to loss after deep downsampling. Secondly, the highly concentrated spatial distribution and severe overlapping of targets frequently result in detection omissions. Thirdly, cluttered background conditions coupled with noise perturbations significantly degrade the feature representation of small objects. In addition, the limited computational power and energy consumption of UAV platforms require detection models to balance accuracy with lightweight deployability.

Currently, single-stage detectors represented by the YOLO series have become an important approach for object detection in UAV scenarios due to their high speed and ease of deployment [4]. YOLOv8 [5] performs excellently, in general, object detection tasks; however, when directly applied to UAV small-object scenarios, it still exhibits deficiencies: its default detection head only utilizes P3–P5 feature layers, resulting in insufficient utilization of high-resolution information for extremely small objects; the static upsampling in the neck network easily causes distortion of fine feature details.

Furthermore, a less-discussed yet critical challenge in multi-scale feature fusion is cross-scale semantic conflict. In UAV aerial imagery, feature maps at different pyramid levels encode information of fundamentally different semantic granularities: shallow layers capture fine-grained spatial details associated with small targets, while deep layers encode high-level semantic context dominated by large background regions. When these heterogeneous features are fused via simple concatenation or element-wise summation—as is standard in PANet-based architectures—the background-dominated semantic responses from deep feature maps tend to suppress the spatially precise but semantically sparse representations of small targets in shallow layers. This phenomenon, termed semantic conflict, can significantly degrade detection recall and localization accuracy for small objects. Addressing semantic conflict through explicit semantic alignment—which adaptively coordinates spatial and semantic inconsistencies across different scales—therefore constitutes an essential design consideration for robust UAV small-object detection.

To tackle these challenges, this study presents ADA-YOLO, an Adaptive Dynamic Aggregation detection network built upon the YOLOv8 architecture. By incorporating a high-resolution P2 detection head, a DySample dynamic upsampling module, alongside an ASFF adaptive spatial feature fusion mechanism acting as the core semantic alignment strategy, the proposed approach significantly bolsters the model’s capacity to identify minute targets within intricate aerial environments. Empirical evaluations executed on the VisDrone2019 and AI-TOD datasets reveal that ADA-YOLO substantially elevates small-object detection efficacy in drone-based applications, achieving this with manageable increments in both parameter count and computational overhead. The primary contribution of this research lies in the efficient and synergistic combination of established modules to specifically address the unique challenges of UAV small-object detection. Rather than proposing isolated components, we designed a cohesive architecture where each module mitigates a distinct bottleneck in small-target representation. The specific combinatorial improvements are summarized below:

1. High-resolution spatial representation via the P2 detection head: By incorporating a feature branch with a stride of 4 into the detection head, we explicitly compensate for the spatial information loss inherent in deep downsampling, significantly strengthening the localization capability for extremely small objects.
2. Content-aware feature reconstruction via DySample: We replace traditional static interpolation with a lightweight dynamic upsampling mechanism. Within our multi-scale architecture, this adaptively enhances target contour sharpness without imposing additional computational burden.
3. Cross-scale semantic alignment via ASFF: To resolve the semantic conflicts exacerbated by the addition of the P2 branch, we utilize pixel-level adaptive weighting for multi-scale feature fusion. This explicitly suppresses cross-scale background noise interference under complex UAV perspectives.

## 2. Related Work

### 2.1. Research on UAV Object Detection

The paradigm of UAV object detection has transitioned from traditional manual feature extraction techniques, including HOG and SIFT, to advanced detectors driven by deep learning. In contrast to two-stage architectures like Faster R-CNN, single-stage frameworks within the YOLO family present greater appeal for drone implementation, as they yield a superior balance between computational speed and detection precision. Nevertheless, the efficacy of generic models remains constrained in aerial environments, primarily attributable to diminutive target scales, crowded spatial arrangements, and chaotic backgrounds [6,7].

While contemporary detection algorithms demonstrate encouraging outcomes on conventional datasets, the inherent attributes of UAV-captured imagery—such as miniature target proportions, highly concentrated distributions, and intricate surroundings—continue to restrict the performance of universal detection architectures in these specific contexts. Therefore, devising structural enhancements customized for UAV small-object detection has emerged as a critical research trajectory.

### 2.2. Related Methods for Small Object Detection

To tackle the challenge of inadequate feature representation inherent to small targets, the current literature has predominantly concentrated on three principal directions: high-resolution feature utilization, feature reconstruction, and multi-scale fusion. Feature Pyramid Networks (FPN) and their improved variants enhance multi-scale detection capability by fusing shallow detailed information with deep semantic information [8,9]. However, for extremely small objects, it is often still necessary to introduce higher-resolution shallow feature branches to strengthen detail representation [10].

Regarding feature reconstruction, while conventional nearest-neighbor and bilinear interpolation techniques provide computational efficiency, they suffer from a lack of content awareness and frequently induce edge blurring. Recently, dynamic upsampling approaches, including CARAFE and DySample, have elevated the quality of feature restoration by adaptively modulating the sampling mechanism. In the area of multi-scale fusion, adaptive fusion methods such as ASFF dynamically weight features at different scales [11], effectively mitigating semantic conflicts that arise from simple concatenation or summation.

In summary, while current approaches have achieved notable advancements in identifying small targets, shortcomings persist regarding the preservation of high-resolution details, the caliber of feature reconstruction, and adaptive multi-scale fusion. Motivated by these limitations, this research proposes specific modifications to the YOLOv8 architecture to augment its capacity for small-object detection within drone-based operational contexts.

## 3. Methodology

### 3.1. Overall Model Architecture

This study utilizes YOLOv8n as the baseline model. The intrinsic architecture of YOLOv8 is fundamentally comprised three primary modules: the Backbone, the Neck, and the Head. The Backbone progressively extracts multi-scale features through modules such as Conv, C2f, and SPPF. The Neck employs a simplified PANet structure to perform both top-down and bottom-up fusion of features from different levels. The Head conducts decoupled detection on the fused features to predict object categories and bounding boxes.

Although YOLOv8 performs well on general object detection, its default design is still suboptimal for UAV small-object scenarios in three aspects: insufficient use of P2 high-resolution features [12,13], detail loss caused by static upsampling, and semantic conflicts in direct multi-scale fusion [14].

**Figure 1:**
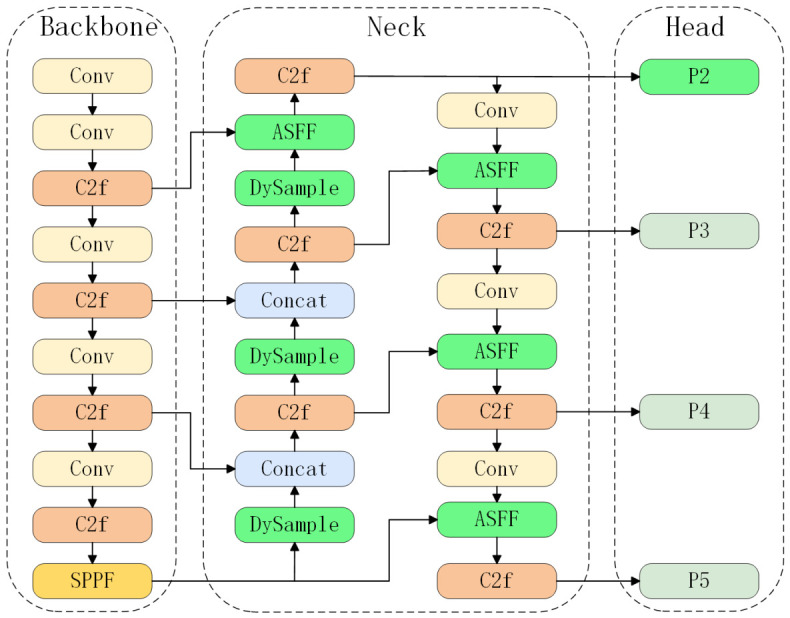
ADA-YOLO Model Architecture, The green blocks indicate the components introduced or modified in the proposed architecture.

To resolve the aforementioned limitations, this study introduces the ADA-YOLO architecture. The comprehensive network topology is depicted in Figure 1, where the green modules represent the components newly added or replaced compared to the baseline YOLOv8. In ADA-YOLO, while preserving the efficient feature extraction proficiency of the Backbone, we optimize the Neck and Head as follows. First, high-resolution P2 shallow features are extracted and a dedicated P2 detection branch is added to the Head. Second, the conventional Upsample modules within the Neck are superseded by DySample dynamic upsampling units. Finally, before multi-scale features are aggregated across layers and fed into the C2f modules for further processing, the ASFF adaptive spatial feature fusion modules are inserted. This design constructs an adaptive dynamic aggregation network with strong sensitivity to small objects.

### 3.2. High-Resolution P2 Detection Head

In deep convolutional networks, there exists an inherent trade-off between the receptive field size and the spatial resolution of feature maps, making it difficult to optimize both simultaneously. As the downsampling stride increases, the receptive field of individual neurons expands exponentially, which helps the model capture global semantic information. However, fine-grained spatial localization information decays rapidly at the same time, exerting a detrimental effect on small object detection.

For UAV images captured at altitudes of several hundred meters, the physical pixel sizes of numerous ground vehicles or pedestrians are often smaller than 10×10. In the native YOLOv8 architecture, under the current 640×640 input setting, the highest-resolution prediction feature layer in the native YOLOv8 head is P3, with a stride of 8 and a spatial size of 80×80. A pedestrian spanning an 8×8 pixel area within the input image is projected down to merely a single 1×1 pixel representation on the P3 feature map. If the target size is further reduced to 4×4 pixels, its mapping may occupy less than one complete pixel, making it difficult for convolutional kernels to capture effective edge and texture information. This severely constrains the accuracy of bounding box regression.

**Figure 2:**
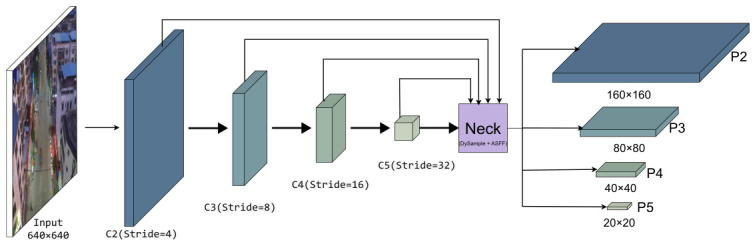
Add a P2 detection branch.

To alleviate the above issues, this paper introduces high-resolution features from the preliminary stages of the Backbone into multi-scale fusion. Specifically, after the completion of feature extraction at the second stage of the Backbone, the shallow-layer features with a stride of 4 and a resolution of 160×160 are explicitly introduced into the Neck network and fused with high-level semantic information across layers. As shown in Figure 2, the improved network architecture adds a dedicated P2 detection branch on top of the original structure, thereby constructing a four-scale prediction system comprising P2, P3, P4, and P5 [15].

By incorporating the high-resolution P2 feature map, the model’s capacity to perceive minute targets is substantially augmented. On the P2 layer (160×160), small objects that originally occupy only 8×8 pixels in the input image are mapped to a 2×2 feature response region. This provides the decoupled detection head with more sufficient local texture information and geometric structural features for both the classification and regression branches, effectively overcoming the resolution bottleneck in extremely small object detection.

While the integration of the P2 branch increases the feature map size to a certain extent, thereby incurring a modest additional cost in terms of parameters and computational complexity, this overhead is acceptable and offers a high cost-performance ratio compared to the substantial improvements achieved in small-object recall and localization accuracy.

### 3.3. DySample Dynamic Upsampling Mechanism

In the top-down construction of the feature pyramid, high-level semantic features need to be fused with low-level high-resolution features through upsampling operations. However, traditional upsampling techniques rely on fixed sampling rules and cannot adaptively model input content. Nearest-neighbor interpolation, which uses the closest pixels, is computationally efficient but produces obvious block artifacts, while bilinear interpolation reduces discontinuity, it still cannot adequately restore high-frequency details.

To achieve higher-quality feature reconstruction, this paper introduces the DySample module at all critical upsampling positions. Unlike more complex methods such as CARAFE and FADE that rely on dynamic convolutional kernel generation [16,17], DySample models the upsampling process as a content-aware spatial sampling location prediction problem [18]. It achieves efficient and adaptive feature resampling by directly learning spatial offsets of sampling points, thereby significantly reducing computational complexity and memory overhead while maintaining strong expressive power.

**Figure 3:**
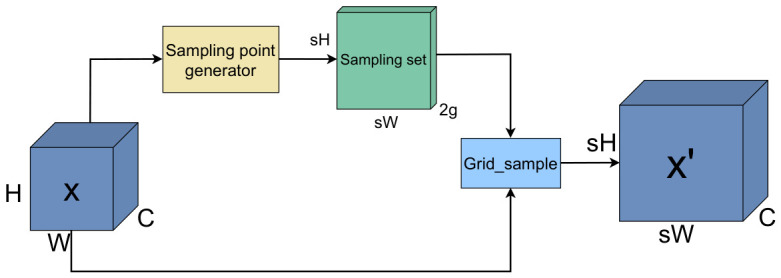
Workflow of the DySample module.

The specific workflow of DySample is shown in Figure 3. The module takes an input feature $X\in R^{C\times H\times W}$ as input and employs a lightweight sampling point generator to predict a set of sampling locations *s* in the high-resolution space. Subsequently, the grid_sample operation is applied to resample the input features, generating the upsampled feature map $X^{\prime }\in R^{C\times sH\times sW}$.

The sampling point generator derives spatial offsets at every spatial location utilizing a linear projection (typically implemented as a 1×1 convolution) of the input features. To elevate the representational capacity whilst regulating computational overhead, the feature channels are partitioned into *g* distinct groups, where each group relies on a shared configuration of sampling offsets.

Mathematically, the offset tensor is first generated as follows:

(1) $$O=\mathrm{Linear}\left(X\right)\in R^{2gs^{2}\times H\times W}$$

where *O* contains the microscopic coordinate offsets in the *x* and *y* directions for each spatial position. Subsequently, the pixel shuffle operation is applied to spatially rearrange the $2s^{2}$ offset dimensions, reconstructing them into the target high-resolution spatial dimensions:

(2) $$O_{\mathrm{reshaped}}=\mathrm{PixelShuffle}\left(O\right)\in R^{2g\times sH\times sW}$$

Meanwhile, the algorithm generates a standard regular high-resolution sampling grid $G\in R^{2\times sH\times sW}$, whose initial coordinate distribution follows that of traditional bilinear interpolation. By superimposing the learned offsets onto this regular grid, the final set of adaptive sampling locations is obtained:

(3) $$S=G+O_{\mathrm{reshaped}}$$

Finally, the highly optimized grid_sample operation in deep learning frameworks is utilized to perform bilinear interpolation-based resampling on the input feature *X*, yielding the upsampled result:

(4) $$X^{\prime }=\text{grid\_sample}\left(X,S\right)$$

The core benefit of DySample for small object detection lies in its content-aware characteristic. In UAV images, when dealing with extremely small-scale vehicles or pedestrians, the spatial offsets *O* learned by DySample adaptively guide the sampling points toward the salient boundaries or high-frequency texture regions of the objects. This not only prevents the forced diffusion of background noise but also markedly amplifies the contour clarity of diminutive targets and the discriminative capacity of the features subsequent to the upsampling procedure. Moreover, since the method relies solely on lightweight 1×1 convolution and interpolation operations, its additional computational overhead is extremely low. It thus serves as a key module in ensuring that the ADA-YOLO model maintains efficient inference performance while improving accuracy.

### 3.4. ASFF Adaptive Spatial Feature Fusion Strategy

After introducing the P2 branch and the high-quality DySample upsampling, the network forms a four-scale feature pyramid (P2, P3, P4, and P5) in the Neck network. However, traditional PANet typically employs static fusion methods such as channel concatenation or element-wise addition during cross-scale feature fusion, implicitly assuming that features at different scales contribute equally at each spatial location.

This assumption does not hold under the UAV perspective. Since the image often contains both large background regions and extremely small-scale objects simultaneously, when shallow detailed features (e.g., P2) and deep semantic features (e.g., P5) are fused indiscriminately at the same spatial position, the background responses in the deep features tend to overwhelm the shallow target information. This phenomenon induces cross-scale semantic conflict, ultimately degrading small object detection performance.

**Figure 4:**
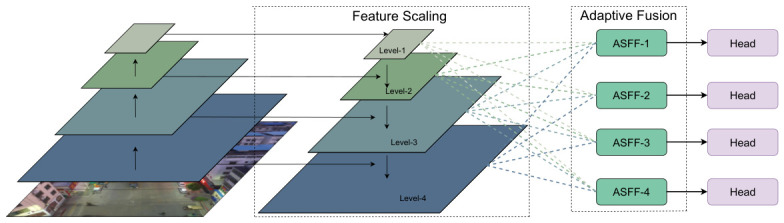
Diagram of the ASFF structure.

To address this issue, this paper introduces the ASFF (Adaptive Spatial Feature Fusion) module to perform scale-wise adaptive fusion of the four-scale features [19]. As shown in Figure 4, each output scale is assigned an independent ASFF module (ASFF-1 to ASFF-4), which is responsible for generating the input features for the corresponding detection head.

Combining the structure shown in the figure, the computation process of ASFF can be divided into two stages: Feature Scaling and Adaptive Fusion.

**(1) Feature Scaling.** For a target output layer l∈{2,3,4,5}, the corresponding ASFF module receives features from four scales $\left\{X^{2},X^{3},X^{4},X^{5}\right\}$ and maps them uniformly to scale *l*:

- When the input feature resolution is higher than the target layer (n<l), downsampling is performed using a stride-2 convolution or pooling operation;
- When the input feature resolution is lower than the target layer (n>l), channel alignment is first conducted via a 1×1 convolution, followed by DySample dynamic upsampling to restore the spatial resolution.

After the above processing, the aligned feature set $\left\{X^{2\to l},X^{3\to l},X^{4\to l},X^{5\to l}\right\}$ is obtained.

**(2) Adaptive Fusion.** At each spatial location (i,j), ASFF generates weights corresponding to the four scales through a lightweight convolutional network:

$$\lambda_{\alpha }^{l}\left(i,j\right),\lambda_{\beta }^{l}\left(i,j\right),\lambda_{\gamma }^{l}\left(i,j\right),\lambda_{\delta }^{l}\left(i,j\right)$$

These weights are then normalized via the Softmax function to obtain:

$$\alpha_{ij}^{l},\beta_{ij}^{l},\gamma_{ij}^{l},\delta_{ij}^{l}$$

where the weights satisfy $\alpha_{ij}^{l}+\beta_{ij}^{l}+\gamma_{ij}^{l}+\delta_{ij}^{l}=1$. Finally, the output feature at layer *l* is given by

(5) $$y_{ij}^{l}=\alpha_{ij}^{l}\cdot x_{ij}^{2\to l}+\beta_{ij}^{l}\cdot x_{ij}^{3\to l}+\gamma_{ij}^{l}\cdot x_{ij}^{4\to l}+\delta_{ij}^{l}\cdot x_{ij}^{5\to l}$$

## 4. Experiments

### 4.1. Datasets and Experimental Setup

The proposed model was evaluated on two publicly available and widely used datasets for aerial and tiny object detection:

1. VisDrone2019 [20]: These dataset features highly diverse urban aerial perspectives and defines 10 object categories with distinct characteristics under UAV top-down viewpoints.
2. AI-TOD [21]: A specialized dataset designed for tiny object detection in aerial imagery, where the average object size is only approximately 12.8 pixels. It is specifically constructed to address the challenges of extremely small object detection in remote sensing scenarios.

The experiments were conducted using an NVIDIA GeForce RTX 3090 GPU with 24 GB of memory (NVIDIA Corporation, Santa Clara, CA, USA). The software environment consisted of CUDA (v11.8), Python (v3.9), PyTorch (v2.0.1), and Ultralytics YOLOv8 (v8.0.180). To ensure reproducibility, the hyperparameter configuration is detailed as follows. Optimization of the network was achieved via the Stochastic Gradient Descent (SGD) algorithm, chosen for its generally stable generalization performance in YOLO-based object detection tasks. Prior to processing, all input imagery was rescaled to dimensions of 640 × 640 pixels, combined with a batch size configuration of 8. This batch size was set to balance training efficiency with the memory limits of the GPU, considering the added high-resolution P2 detection head. An initial learning rate of 0.01 was established, aligning with standard YOLOv8 configurations, which subsequently underwent decay governed by a cosine annealing schedule. This schedule facilitates a smooth learning rate transition, helping to mitigate convergence instability often encountered in complex UAV scenes. The comprehensive training phase spanned a duration of 300 epochs, as empirical observations indicated sufficient convergence without noticeable overfitting within this timeframe. To expedite the convergence process, the network was initialized utilizing the official yolov8n.pt pre-trained weight parameters supplied by Ultralytics. By detailing these specific settings, the experimental results can be reliably reproduced based on standard YOLO implementations.

Regarding the quantitative assessment of algorithmic efficacy, this research implements conventional metrics universally acknowledged within the object detection domain. The structural complexity of the framework is quantified through its parameter count and floating-point operations (FLOPs), which delineate spatial and computational demands, respectively. Furthermore, to assess the model’s operational efficiency and real-time processing capability—a critical requirement for UAV deployment—inference speed is measured in Frames Per Second, (FPS). To gauge detection precision, the mean Average Precision (mAP) serves as the principal evaluative criterion. Specifically, mAP@0.5 signifies the mean average precision at a designated Intersection over Union (IoU) threshold of 0.5, whereas mAP@0.5:0.95 reflects the integrated average of mAP scores derived across an IoU continuum extending from 0.5 to 0.95, computed at incremental intervals of 0.05.

### 4.2. Experimental Results

#### 4.2.1. Experimental Results on the VisDrone2019 Dataset

Table 1 delineates the empirical findings evaluated on the VisDrone2019 test set. It is evident that the introduced ADA-YOLO framework realizes substantial enhancements relative to the baseline YOLOv8n architecture. Notably, it secures the highest mAP@0.5:0.95 among all comparative techniques documented in Table 1, whilst simultaneously sustaining a robust mAP@0.5 performance. In quantitative terms, the mAP@0.5 metric experiences an upsurge of 11.3%, and the mAP@0.5:0.95 is elevated by 8.2%. Experimental results indicate that the introduced adaptive dynamic feature fusion mechanism can effectively enhance multi-scale feature representation capability, particularly yielding substantial gains in detection accuracy under complex scenes. YOLOv8n, YOLO26n [22], and ADA-YOLO were trained under identical experimental settings. Results for other models are cited from their original papers.

**Figure 5:**
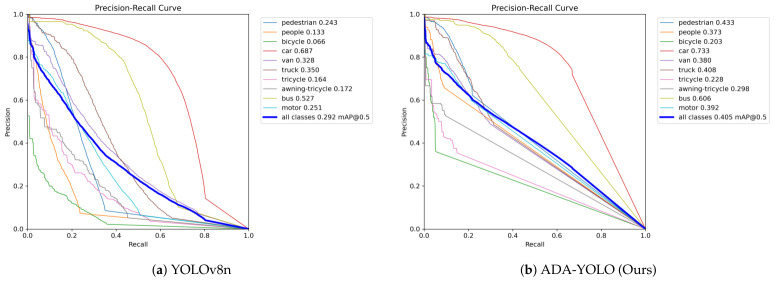
Precision-Recall curves comparison on VisDrone2019.

Moreover, as illustrated in Figure 5, an evaluation of the Precision-Recall (PR) curves for the baseline architecture and the introduced ADA-YOLO on the testing dataset reveals that the trajectory of our approach is noticeably smoother and positioned superior to that of the baseline. This observation signifies that the proposed strategy preserves elevated detection accuracy throughout a diverse spectrum of recall thresholds. Notably, within the moderate-to-high recall intervals, the degradation in precision occurs at a substantially decelerated rate, thereby corroborating the augmented resilience of the model when confronted with intricate environmental conditions.

**Figure 6:**
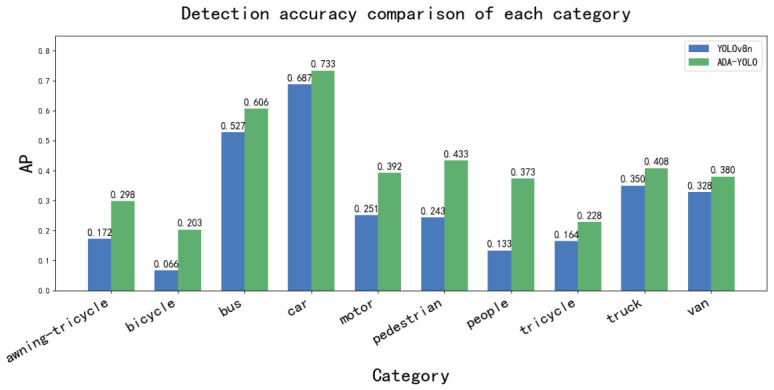
Detection accuracy of YOLOv8n and ADA-YOLO in different categories.

To provide a more granular and quantitative perspective on category-specific performance, Figure 6 visualizes the per-category average precision (AP) comparison between the two models on the VisDrone2019 dataset, while medium and large object categories such as bus and car maintain stable and high-level object localization capabilities, the most substantial performance gains are explicitly concentrated in small and densely distributed categories. Specifically, despite the extreme detection difficulty, ADA-YOLO achieves highly significant improvements over the baseline in challenging categories such as bicycle (+13.7%), pedestrian (+19.0%), and people (+24.0%). These quantitative findings, consistent with the aforementioned PR curve trends, robustly indicate that the synergistic design of the high-resolution P2 detection head and the ASFF adaptive spatial feature fusion mechanism successfully alleviates the issue of small-target representations being readily obscured by background clutter.

**Figure 7:**
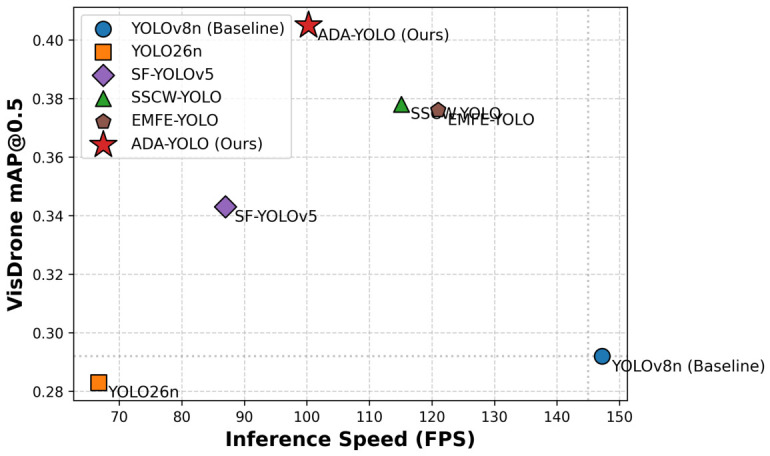
Speed-accuracy trade-off analysis on the VisDrone dataset. The proposed ADA-YOLO achieves the optimal balance between detection accuracy (mAP@0.5) and inference speed (FPS).

Furthermore, to address practical deployment considerations for UAV platforms and provide a comprehensive evaluation, a speed-accuracy trade-off analysis was conducted, as illustrated in Figure 7, while the introduction of dynamic upsampling and adaptive fusion modules introduces a modest computational overhead, ADA-YOLO sustains a rigorous inference speed of 100.23 FPS on the NVIDIA RTX 3090 GPU. This measurement significantly exceeds the standard real-time processing threshold of 30 FPS, confirming that the substantial improvements in small object detection accuracy are achieved without compromising the real-time operational viability of the model.

**Figure 8:**
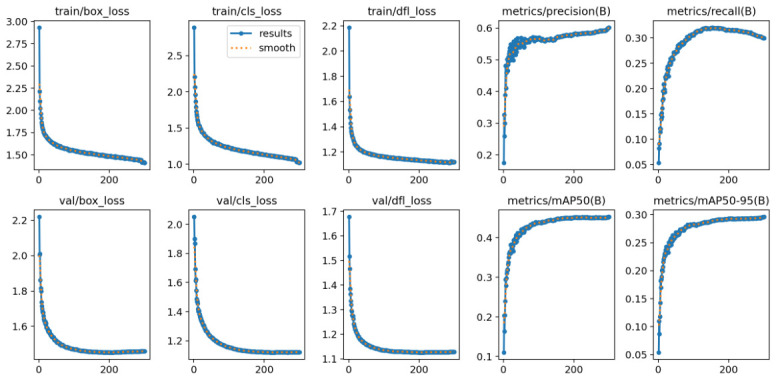
Training curves of the ADA-YOLO model.

As illustrated by the training process curves in Figure 8, ADA-YOLO exhibits excellent convergence characteristics during the training phase. During the initial phases of training, the box loss, cls loss, and dfl loss undergo a swift reduction, progressively reaching a state of stabilization in the concluding epochs. Such a trajectory implies that the architecture efficiently extracts target representations via a steady optimization procedure. Concurrently, both the mAP@0.5 and mAP@0.5:0.95 metrics on the validation set manifest a continuous upward progression until final convergence is achieved, exhibiting no conspicuous indications of performance deterioration or overfitting phenomena.

#### 4.2.2. Experimental Results on the AI-TOD Dataset

To further substantiate the generalization capability of the ADA-YOLO architecture concerning exceptionally minute targets, comparative evaluations were executed utilizing the AI-TOD dataset, which is characterized by an average object dimension of merely 12.8 pixels. The corresponding empirical outcomes are documented in Table 2.

In the AI-TOD dataset, the average absolute size of objects is only approximately 12.8 pixels, making feature attenuation highly likely to occur in deep network layers. Thanks to the ultra-high-resolution receptive field provided by the P2 detection head and the feature reconstruction capability of DySample, ADA-YOLO improves mAP@0.5 by 3.4% and mAP@0.5:0.95 by 4.5% compared to the baseline model, demonstrating strong capability in capturing extremely tiny objects.

### 4.3. Ablation Study

To evaluate the isolated impacts of the three primary novel components within ADA-YOLO on the comprehensive architecture, as well as their mutual enhancements, ablation studies were performed utilizing the VisDrone2019 dataset. The integration of the P2 detection head is denoted as A, the application of the DySample unit as B, and the incorporation of the ASFF mechanism as C. Through the sequential accumulation of these advanced modules, it becomes possible to systematically substantiate their independent efficacies and combined advantages. The resultant data are synthesized in Table 3:

From the ablation study, it can be observed that the introduction of the high-resolution P2 branch significantly enhances the representation capability for extremely small objects, achieving a 5.3% improvement in mAP@0.5 with only approximately 2.1 G additional FLOPs. Further replacing the upsampling operation with DySample yields an additional 1.8% accuracy gain with almost no increase in computational cost, and produces synergistic benefits when combined with the P2 branch. The introduction of the ASFF module further strengthens the feature fusion performance of the model, delivering a standalone improvement of 3.3% in accuracy and effectively suppressing shallow-layer noise within the multi-scale structure. Ultimately, the ADA-YOLO model, constructed through the synergistic integration of P2, DySample, and ASFF, strikes an optimal equilibrium between detection accuracy and computational efficiency, yielding an mAP@0.5 score of 0.405 and culminating in a substantial enhancement of comprehensive performance.

Furthermore, regarding the experimental setup, it should be noted that all models evaluated in this ablation study were strictly initialized with identical COCO pre-trained weights. This methodological choice aligns with the standard deployment paradigms of lightweight detectors in real-world UAV applications, where transfer learning is essential to establish baseline feature extraction in highly complex, small-object dense scenarios. By maintaining this uniform prior knowledge as a strictly controlled variable throughout the ablation process, we ensure that the observed substantial enhancement in comprehensive performance is directly and solely attributable to the synergistic integration of the ADA-YOLO architecture, rather than any biased initialization.

### 4.4. Comparison of Detection Results

To more intuitively demonstrate the performance advantages of ADA-YOLO over the baseline YOLOv8 model, and to provide a comprehensive evaluation, we have expanded our visual comparison of detection results across multiple challenging scenarios. We selected representative images from both the VisDrone2019 and AI-TOD datasets, encompassing typical UAV imagery challenges such as extremely dense small objects, severe occlusions, high-altitude shooting perspectives, and low-contrast backgrounds.

**Figure 9:**
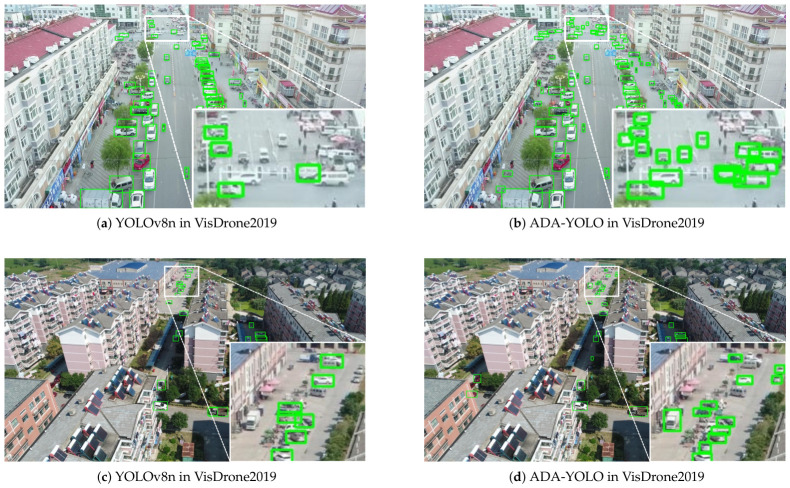
Visualization of detection results on the VisDrone2019 dataset with zoomed-in details. (**a**) YOLOv8n in Scene 1; (**b**) ADA-YOLO in Scene 1; (**c**) YOLOv8n in Scene 2; (**d**) ADA-YOLO in Scene 2.

Figure 9 illustrates two distinct urban scenarios from the VisDrone2019 dataset, where targets are densely packed. To better highlight the performance differences, we have enlarged specific regions containing extremely small targets in the distant background. In the first scene (Figure 9a,b), observing a busy intersection, the baseline YOLOv8n model exhibits noticeable missed detections when facing distant, clustered vehicles. In contrast, ADA-YOLO detects more of these distant targets whose features are less obvious. In the second scene (Figure 9c,d), focusing on a residential parking area characterized primarily by distant and overlapping targets, along with partial occlusions from trees and shadows, ADA-YOLO shows a slight but effective improvement by retrieving several clustered vehicles that the baseline missed. Benefiting from the high-resolution P2 detection head and the content-aware DySample module, ADA-YOLO demonstrates a more robust fine-grained spatial feature representation capability in cluttered urban environments.

**Figure 10:**
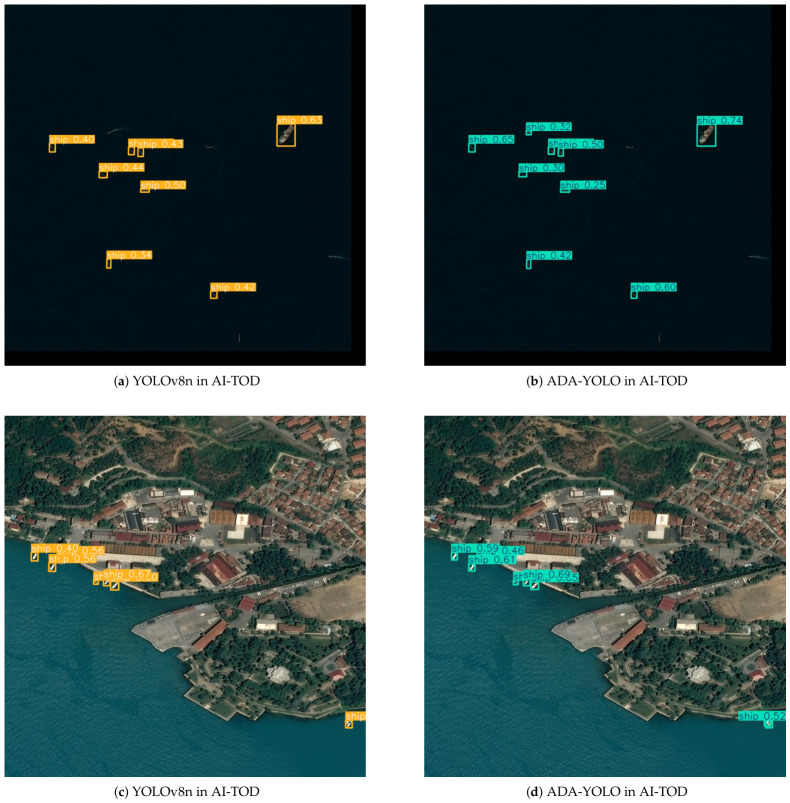
Visualization of detection results on the AI-TOD dataset. (**a**) YOLOv8n in Scene 1; (**b**) ADA-YOLO in Scene 1; (**c**) YOLOv8n in Scene 2; (**d**) ADA-YOLO in Scene 2.

To further validate the model’s capability on extremely small objects, Figure 10 presents two different maritime scenarios from the AI-TOD dataset. The first scene (Figure 10a,b) features a dark, low-contrast ocean background where the target ships blend into the water surface and occupy very few pixels, while YOLOv8n misses several smaller, fainter ships, ADA-YOLO is able to capture more of these targets with less discernible features. The second scene (Figure 10c,d) displays a coastal area where ships are tightly clustered near the shoreline. Compared to the baseline’s struggle to distinguish the densely packed targets in the complex shore environment, ADA-YOLO exhibits stronger feature extraction capabilities. By utilizing the Adaptive Spatial Feature Fusion (ASFF) mechanism to suppress background noise interference, ADA-YOLO effectively reduces missed detections among these challenging tiny ships and provides more stable bounding boxes.

Overall, these expanded visualization outcomes consistently align with our quantitative empirical findings. They provide visual evidence that the proposed ADA-YOLO effectively mitigates the issue of missing dense and minute objects in various complex UAV aerial imagery scenes, achieving more reliable detection performance than the baseline model.

## 5. Conclusions and Future Work

### 5.1. Conclusions

This research addresses the formidable challenges associated with small object detection in Unmanned Aerial Vehicle (UAV) imagery, encompassing diminutive target scales, dense spatial distributions, and intricate backgrounds. Based on the YOLOv8n baseline model, we propose an Adaptive Dynamic Aggregation network named ADA-YOLO. The core improvements include:

- Introduction of a high-resolution P2 detection head to construct a P2–P5 four-scale prediction structure, thereby enhancing the fine-grained spatial information representation for extremely small objects;
- Adoption of DySample dynamic upsampling to replace traditional static interpolation, improving feature reconstruction quality;
- The incorporation of the ASFF adaptive spatial feature fusion mechanism during the multi-scale integration phase to mitigate semantic discrepancies and suppress background noise interference.

Empirical evaluations substantiate that the ADA-YOLO architecture attains substantial performance enhancements across both the VisDrone2019 and AI-TOD datasets. On VisDrone2019, mAP@0.5 increases from 0.292 to 0.405, and mAP@0.5:0.95 increases from 0.167 to 0.249. On AI-TOD, mAP@0.5 rises from 0.410 to 0.444, and mAP@0.5:0.95 rises from 0.176 to 0.221. The model has only 3.17M parameters and 13.2G FLOPs, maintaining a favorable accuracy-complexity balance while substantially improving detection precision. Ablation experiments validate the individual effectiveness of each module and their synergistic gains. Visualization results further show that ADA-YOLO effectively reduces missed detections, improves detection confidence, and enhances localization accuracy. Overall, the proposed method provides a practical solution for small object detection in UAV scenarios.

### 5.2. Future Work

Future research endeavors will concentrate on three principal directions: First, deploying the model on edge hardware such as Jetson to systematically verify its real-time applicability in terms of inference latency, frame rate, power consumption, and model compression effects; Second, extending the model to more cross-scene, cross-weather, and cross-altitude datasets as well as real-world UAV mission environments to validate its generalization ability, and further improving detection stability by combining fine-grained label assignment strategies, rotated bounding box detection mechanisms, and temporal sequence modeling to address challenges like dense occlusion, extreme scale variations, and rotated objects in UAV imagery; Third, introducing semi-supervised or self-supervised learning along with targeted data augmentation strategies to reduce the reliance on large-scale manually annotated samples and enhance the model’s applicability under low-labeling-cost conditions.

## Figures and Tables

**Table 1:** Performance comparison of different detection models on the VisDrone2019 dataset, ^†^ Cited from original paper with author-reported configuration.

Model	Param (M)	FLOPs (G)	FPS	${\mathrm{mAP}}_{50}^{\mathrm{test}}$	${\mathrm{mAP}}_{50:95}^{\mathrm{test}}$
YOLOv8n	3.01	8.2	147.24	0.292	0.167
YOLO26n	2.51	5.8	66.71	0.283	0.164
SF-YOLOv5 [23]	2.24	13.8	86.96	0.343 ^†^	0.182 ^†^
HIC-YOLOv5 [24]	8.39	–	–	0.369 ^†^	0.208 ^†^
SSCW-YOLO [25]	2.73	8.7	115.10	0.378 ^†^	0.223 ^†^
YOLO-MARS [26]	2.93	–	–	0.409 ^†^	0.234 ^†^
EMFE-YOLO [13]	3.00	33.1	121.0	0.376 ^†^	–
ADA-YOLO (Ours)	3.17	13.2	100.23	0.405	0.249

**Table 2:** Performance comparison of different models on the AI-TOD dataset.

Model	Param (M)	FLOPs (G)	FPS	${\mathrm{mAP}}_{50}^{\mathrm{test}}$	${\mathrm{mAP}}_{50:95}^{\mathrm{test}}$
YOLOv8n	3.01	8.2	147.24	0.410	0.176
FMFN-YOLO [27]	8.6	47.1	158.73	0.436	0.201
MPE-YOLO [28]	8.7	4.4	71	0.471	0.218
FBRT-YOLO-S [29]	2.9	22.9	142	0.458	0.202
ADA-YOLO (Ours)	3.17	13.2	100.23	0.444	0.221

**Table 3:** Ablation study of ADA-YOLO on the VisDrone2019 dataset. The symbols ✓ and × indicate that the corresponding module is included and not included, respectively.

Model	A	B	C	Param (M)	FLOPs (G)	${\mathrm{mAP}}_{50}^{\mathrm{test}}$	${\mathrm{mAP}}_{50:95}^{\mathrm{test}}$
Baseline	×	×	×	3.01	8.2	0.292	0.167
+ A	✓	×	×	3.05	12.4	0.345	0.198
+ B	×	✓	×	3.03	8.7	0.310	0.183
+ C	×	×	✓	3.10	8.4	0.325	0.191
+ A + B	✓	✓	×	3.08	13.1	0.368	0.216
+ A + C	✓	×	✓	3.14	12.7	0.381	0.228
+ A + B + C	✓	✓	✓	3.17	13.2	0.405	0.249

## Data Availability

Publicly available datasets were analyzed in this study. These datasets can be found on the official VisDrone2019 and AI-TOD dataset webpages. Additional experimental data are available from the corresponding author upon reasonable request.
